# Tolerogenic IDO^+^ Dendritic Cells Are Induced by PD-1-Expressing Mast Cells

**DOI:** 10.3389/fimmu.2016.00009

**Published:** 2016-01-25

**Authors:** Cecilia Pessoa Rodrigues, Ana Carolina Franco Ferreira, Mariana Pereira Pinho, Cristiano Jacob de Moraes, Patrícia Cruz Bergami-Santos, José Alexandre Marzagão Barbuto

**Affiliations:** ^1^Laboratory of Tumor Immunology, Department of Immunology, Institute of Biomedical Sciences, University of Sao Paulo, São Paulo, Brazil; ^2^Center for Cellular and Molecular Studies and Therapy (NETCEM), University of Sao Paulo, São Paulo, Brazil

**Keywords:** mast cells, tolerogenic DCs, Tregs, IDO, STAT-3

## Abstract

Mast cells (MCs) are tissue resident cells, rich in inflammatory mediators, involved in allergic reactions, and with an increasingly recognized role in immunomodulation. Dendritic cells (DCs), on the other hand, are central to the determination of immune response patterns, being highly efficient antigen-presenting cells that respond promptly to changes in their microenvironment. Here, we show that direct cell contact between immature monocyte-derived DCs (iDCs) and MC bends DCs toward tolerance induction. DCs that had direct contact with MC (MC-iDC) decreased HLA-DR but increased PD-L1 expression and stimulated regulatory T lymphocytes, which expresses FoxP3^+^, secrete TGF-β and IL-10, and suppress the proliferation of mitogen-stimulated naïve T lymphocytes. Furthermore, MC-iDC expressed higher levels of indoleamine-2,3-deoxigenase (IDO), a phenomenon that was blocked by treatment of MC with anti-PD-1 or by the treatment of DCs with anti-PD-L1 or anti-PD-L2, but not by blocking of H1 and H2 histamine receptors on DCs. Contact with MC also increased phosphorylated STAT-3 levels in iDCs. When a STAT-3 inhibitor, JSI-124, was added to the DCs before contact with MC, the MC-iDC recovered their ability to induce allogeneic T cell proliferation and did not increase their IDO expression.

## Introduction

Mast cells (MCs) are myeloid cells that undergo their final differentiation after migration to skin, mucosa, or serosa cavities, where they represent a heterogeneous population. In these tissues, immature MCs recruited from circulation mature and reside in proximity to blood vessels, nerves, smooth muscle, mucus-producing glands, and hair follicle (1). These niches share the common feature of allowing the prompt exposure of MC to environmental stimuli, such as allergens and pathogens (2, 3). Thus, MCs are among the first cells to interact with antigens and irritants, having a decisive role, along with dendritic cells (DCs), in promoting inflammation and modulating the adaptive immune response (4–6). As, for example, in asthma, where MCs and DCs cooperate *in vivo*, through IFN-γ production (7), to promote inflammation (8).

In contrast to their proinflammatory role in allergic disorders, an essential MC role in the induction of immune tolerance has been described in mice (9). In a skin-graft model, donor-specific transfusion-induced tolerance was associated with graft infiltration by MC and regulatory T cells (Tregs) and could not be induced in mice lacking MC (10). On the one hand, in allergic reactions, where MC are associated with tissue lesions (11), the existence of such a regulatory circuit, where MC infiltration would decrease local immune responses, and thus, restrict tissue damage, would be beneficial to the organism. On the other hand, in conditions such as tumors, where MC infiltration is also frequently described (12, 13), the presence of MC-decreasing local immune responses would be detrimental and could contribute to the immune evasion, which is the characteristic of tumors.

However, the mechanisms through which MCs contribute to tolerance induction are not clearly defined. MCs may directly induce regulatory differentiation of T cells (10) or migration of immature DCs to lymph nodes (9, 14), and here, we describe yet another mechanism: the increase in indoleamine-2,3,-deoxigenase (IDO) expression by immature DCs after contact with MC. We demonstrate that this increase depends on the interaction of the programmed cell death-1 molecule (PD-1), expressed by MC, and its DC-expressed ligands, PD-L1 and PD-L2. Finally, we present evidence implicating STAT-3 and the non-canonical NF-kB pathway in the MC-induced modification of DCs associated with their contact-induced ability to activate regulatory T cells (Tregs).

## Materials and Methods

### Sample Collection and Cell Separation

This study was approved by the Institutional Ethics Committee (ICB-USP 275.307/CEP). After informed consent, peripheral blood mononuclear cells (PBMC) were obtained from healthy donors by leukapheresis at the Hospital Alemão Oswaldo Cruz, São Paulo, Brazil. Mononuclear cells were separated over a density gradient (*d* = 1.076) (Ficoll-Paque, Amersham Pharmacia Biotech, Uppsala, Sweden) and suspended in Iscove’s Modified Dulbecco Medium (IMDM), supplemented with 10% fetal calf serum, penicillin (100 U/mL), streptomycin (100 μg/mL), and amphotericin B (0.25 μg/mL Gibco^©^, USA) (I-10).

### *In Vitro* MC Generation

Mast cells were differentiated as described by Saito et al. (15), with modifications. Briefly, CD34^+^ cells from peripheral blood were isolated by positive immunomagnetic separation and cultured in 24-well plates in 100 μL of METHOCULT™ (Stem Cell) plus 200 μL of IMDM, supplemented with stem cell factor (SCF), Interleukin (IL)-6, and IL-3 (200, 50, and 5 ng/mL, respectively) per well. After 2 weeks, 100 μL of METHOCULT™ (Stem Cell) plus 200 μL of IMDM supplemented with SCF and IL-6 (200 and 50 ng/mL, respectively) were added to each well. At week 4, 1 mL of supplemented IMDM (SCF, 200 ng/mL; IL-6, 50 ng/mL; insulin–transferrin–selenium solution, Gibco^©^, catalog no. 41400-045, 100 μL/mL) was added to each well. At week 6, non-adherent cells were transferred to a 12-well plate in supplemented IMDM [SCF, 100 ng/mL; IL-6, 50 ng/mL; insulin–transferrin–selenium solution (20%); 20% of 10% BSA in phosphate-buffered saline]. Two weeks thereafter, non-adherent cells were transferred to six-well plates and cultured with I-10 supplemented with SCF (100 ng/mL) and IL-6 (50 ng/mL); 1 week later, the cells were harvested.

### MC Phenotype Analysis

Cell labeling and flow cytometry acquisition were described previously (16). The cells were labeled for CD13, CD117, PD-1 (Becton Dickinson, San Jose, CA, USA), and FCϵRIα (BioLegend), acquired in a FACSCanto II cytometer (Becton Dickinson, USA) and analyzed using the FlowJo software 8.7.2 (Tree Star). At least 20,000 events in the MC gate, determined by forward (FSC) and side (SSC) scatters, were acquired per sample.

### Monocyte-Derived Dendritic Cells Generation and Coculture with MC

Peripheral blood mononuclear cells from the same donors used for MC generation were thawed, separated over a Ficoll-Paque gradient and seeded in 24-well plates in I-10 (2.5 × 10^6^ cells/mL). After overnight incubation at 37°C, non-adherent cells were removed and GM-CSF and IL-4 (both at 50 ng/mL; PeproTech, Mexico) were added (17). On day 5, immature DCs were obtained, harvested on ice, and cultured in I-10 for further 2 days, either alone (iDCs) or cocultured in direct contact with MC (MC-iDC) in a 5 iDC:1 MC ratio. On day 7, the cells were harvested and their viability (>95%) assessed by trypan blue staining. Alternatively, iDCs were cultured at the bottom of a 24-well transwell plate, which allowed the passage of soluble mediators through a 0.4-μm pore, and MC were seeded in the upper compartment in I-10; DCs thus obtained will be identified as TW-iDCs throughout the experiments.

Antibodies and inhibitors were added to these cocultures as described in each experiment.

### Evaluation of CD107a Expression by CD117^+^ Cells

For the detection of CD107a expression, MC submitted to various culture conditions (in the presence of PMA 100 nM; coculture with iDC; isolated culture) were seeded in a 96-well-plate (1 × 10^5^ MC/well) and after 15 min treated with brefeldin-A (10 μg/mL, BD Pharmingen) and with PE-labeled anti-CD107a. The cells were incubated at 37°C for 12 h, and then harvested, washed with PBS, and labeled with fluorescence-labeled anti-CD11c and anti-CD117. Cells were acquired, at least 20,000 events per gate, in a FACSCanto II cytometer (Becton Dickinson, USA) and analyzed, using the FlowJo software 8.7.2 (Tree Star).

### DC Phenotype Analysis

Cells were stained with fluorescence-labeled antibodies for CD11c, HLA-DR, CD80, CD86, and PD-L1. At least 10,000 events in the DCs (FSC × SSC) gate were acquired per sample. The frequency and median fluorescence intensity (MFI) of the positive cells for each marker were determined within the CD14^−^CD11c^+^ population.

### T Cell Proliferation Assay

Allogeneic CD3^+^ T cells were purified by negative magnetic selection of CD14, CD15, CD16, CD19, CD34, CD36, CD56, CD123, and CD235a-positive cells; the recovered CD3^+^ cells (>95% purity) were used in CFSE dilution assays, as described (16).

### Intracellular Staining

For the analysis of CD3^+^ T lymphocytes, these were harvested, at day 5 of culture, from the various cocultures with DCs, labeled for FoxP3, TGF-β, and IL-10 expression and analyzed by flow cytometry.

The frequency of FoxP3^+^ cells was analyzed using the e-Bioscience Foxp3/Transcription Factor Staining Buffer Set (Affymetrix, e-Bioscience, USA) as described in the manufacturer’s protocol. Before intracellular staining, the cells were labeled with fluorescence-labeled anti-CD4 and anti-CD25 (Becton Dickinson, USA).

The frequency of IL-10- and TGF-β-producing T cells was evaluated using the Protein Transport Inhibitor assay (BD Bioscience, USA); the BD GolgiPlug™ (brefeldin-A) was added to the culture 6 h before harvesting and labeling the cells with fluorescence-labeled anti-CD4, anti-CD25, and for cell viability (Life Technologies). After resuspending the cells in BD Perm/Wash, they were labeled with anti-IL-10 and anti-TGF-β (BD Bioscience, USA).

Dendritic cells and MC were harvested after 16 h of coculture, labeled with anti-CD11c^+^ (DCs) and anti-CD117^+^ (MC), and after brefeldin-A treatment, also labeled for TGF-β and TNF-α. TGF-β production was evaluated by flow cytometry using the Protein Transport Inhibitor (BD Bioscience, USA), after 6 h of brefeldin-A treatment. For TNF-α detection, labeling of the cells was performed after 15 h of brefeldin-A treatment. Data were acquired, at least 20,000 events/gate, in a FACSCanto II cytometer (Becton Dickinson, USA) and analyzed using the FlowJo software 8.7.2 (Tree Star).

### *In Vitro* Suppression Assay

CD3^+^CD4^+^CD25^+^ T Lymphocytes stimulated by the various DC preparations were isolated using the Regulatory T Cell Isolation Kit (Miltenyi Biotec). These cells were added to violet cell tracer (VCT, Life Technologies)-labeled naïve allogeneic T lymphocytes (Naïve CD4^+^ T cells isolation kit, Miltenyi Biotec) and seeded in round-bottom 96-well plates at ratios of 1:1 or 1:5. Phytohemagglutinin-A (PHA – Gibco cat# 10576-015) was added to the wells (1% v:v) and after 72 h at 37°C, the cells were harvested and analyzed (18). Naïve T cell proliferation was evaluated by VCT dilution in live CD3^+^ cells. At least 20,000 events per gate were acquired per sample.

### RT- and qPCR

Total RNA was extracted from CD11c^+^ isolated by anti-FITC microbeads (Miltenyi, no. 130-048-701) using TRIzol (Life Technologies). RNA samples were reverse transcribed to cDNA using SuperScript^®^ III Reverse Transcriptase (Life Technologies) according to the manufacturers’ instructions. Real-time PCR for *IDO1* (F: GTGCAGGCCAAAGCAGCG and R: CCGCAGGCCAGCATCACCT), *SOCS3* (F: TCAAGACCTTCAGCTCCAAG and R: TGACGCTGAGCGTGAAGAAG), *SOCS5* (F: ACCCAGAGTTCATTGGATGC and R: CCCACAGTATCCTGCAACCT), *Rel-B* (F: GATTTGCCGAATTAACAAGGAA and R: GCTGAACACCACTGATATGTCC), *NFkB1* (F: CGTGAAGATGCTGCTGGCCG and R: CCAAGTGCAAGGGCGTCTGGT), *NFkB2* (F: GCTGGGGATCTGCGCCGTTT; R: CGCTGCTCGGCCTCCGTAAG), or β-*actin* (F: CAGGCACCAGGGCGTGATGG and R: CGATGCCGTGCTCGATGGGG) was performed using SYBR green (Life Technologies) set for an initial denaturation step at 95°C for 10 min, amplification with 40 denaturation (95°C, 15 s), annealing (60°C, 15 s), and extension cycles (72°C, 30 s); followed by a final extension at 72°C for 6 min. PCR results were analyzed using the *MXPro3000* software and normalized by β-actin expression.

### Idoleamine-2,3-Deoxigenase Detection by Flow Cytometry

Cells were harvested after 16 h of coculture, washed twice, and labeled for cell viability (Life Technologies), CD11c (IgG1) and CD117 (IgG1) expression (BD Bioscience, San Jose, CA, USA). Next, the cells were permeabilized (with the protocol used for FoxP3 evaluation), mouse serum (2%), and after 15 min at room temperature, anti-IDO (IgG2b) (Abcam, USA) (1 μg/10^6^ cells) were added. After 40 min at 37°C in a humid chamber, the Alexa633-labeled secondary antibody (goat anti-mouse IgG2b, eBiosciences) was added. After another 40 min of incubation, the cells were washed and acquired in a FACSCanto II cytometer (Becton Dickinson, USA). At least 30,000 events per gate were acquired per sample; data were analyzed with the FlowJo software ver. 8.7.2 (Tree Star).

### STAT-3 Phosphorylation Assay

After extracellular staining, DCs were treated with 1% paraformaldehyde and kept for 10 min at room temperature. Next, the cells were washed with PBS and permeabilized by adding 200 μL of ice-cold methanol (90%). These cells were maintained for 30 min at −20°C, then washed with PBS, and labeled with anti-p-STAT-3 (Tyr705) or with the isotype control antibody (BD) for 20 min at 4°C. After that, the cells were washed with PBS and the pellet resuspended in PBS–BSA 0.05%. The cells were, then, analyzed by flow cytometry; at least 20,000 events per DC gate were acquired in a FACSCanto II cytometer (Becton Dickinson, USA) and analyzed using the FlowJo software 8.7.2 (Tree Star).

### Western Blot Analysis

Protein extracts, obtained by RIPA lysis of 1 × 10^6^ CD11c^+^ cells separated by magnetic beads, were transferred onto PVDF membranes (20 μg/well) and probed with antibodies against p-STAT-3 (1:2000, Cell Signaling) or beta-actin (1:30,000, Sigma-Aldrich). Proteins of interest were detected with HRP-conjugated rabbit anti-mouse IgG antibody (1:10,000, Cell Signaling) and visualized with the Pierce ECL Western blotting substrate (Thermo Scientific), according to the manufacturer’s protocol.

### PD-1, PD-L1, and PD-L2 Blocking

Mast cells were treated with anti-PD1 (clone MIH4, BD) (10 μg/10^6^ cells). After 40 min of treatment, the cells were harvested, washed with PBS–BSA 0.1%, and cultured with iDCs.

Dendritic cells were treated either with anti-PDL1 (clone MIH1, e-Bioscience) or anti-PDL2 (clone MIH18, e-Bioscience) (10 μg/10^6^ cells). The cells were incubated at room temperature for 40 min, then were harvested, washed with PBS–BSA 0.1%, counted, and cocultured with MC in a 5:1 ratio.

### JSI-124, Olopatadine Hydrochloride (Anti-H1), or Cimetidine (Anti-H2) Treatments

Dendritic cells, cultured in six-well plates (10^6^ cells/mL), were treated with the STAT-3 inhibitor JSI-124 (Sigma-Aldrich, Saint Louis, MO, USA) (0.5 μM) and incubated at 37°C for 16 h. Afterward, the cells were washed twice with PBS–BSA 0.1%, counted, and cultured with MCs, at the ratio of 5 DCs:1 MC.

In other experiments, before the coculture, DCs were treated either with olopatadine hydrochloride (anti-H1) or cimetidine (anti-H2) (Sigma-Aldrich, Saint Louis, MO, USA) (50 μg/mL) and incubated at 37°C. After 1 h, the cells were washed twice with PBS–BSA 0.1% and cultured with MCs at the ratio of 5DCs:1MC.

### Statistical Analysis

One-way ANOVA with the Tukey’s *post hoc* test or the unpaired Student’s *t*-test were used for comparisons as indicated in each experiment; the GraphPad Prism 6.0 was used for the analyses and statistical significance was set at *p* < 0.05.

## Results

### iDCs Surface Phenotype Is Affected by Both MC Soluble Products and Cell Contact

**Figure 1:**
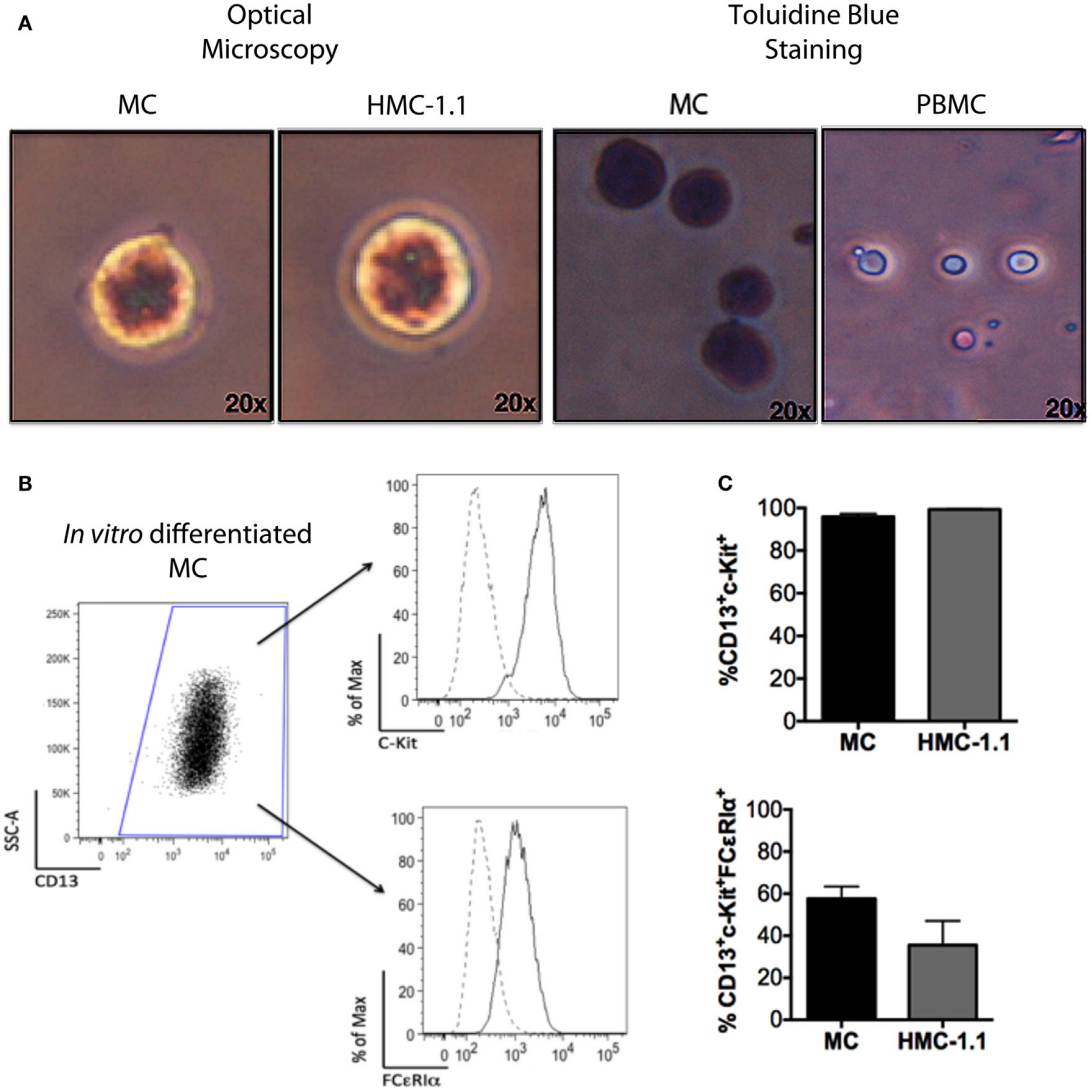
**Phenotype of *in vitro*-differentiated MC and of the HMC-1.1 cell line**. Adult peripheral CD34^+^ cells were isolated using immunomagnetic beads and induced to differentiate into MC, as described. **(A)** Morphology of the *in vitro* cells after 8 weeks, showing toluidine blue staining, similar to that of the human mastocytoma line HMC-1.1. **(B)** Representative flow cytometry analysis showing CD13^+^ cells and within those, c-kit^+^, and FCϵRIα^+^ cells (dotted lines represent unstained cells). **(C)** Comparison of the immunophenotype of *in vitro*-differentiated MC (*n* = 10) and cells of the HMC-1.1 line.

To investigate if MC could affect DCs phenotype and function, we differentiated MC *in vitro* from peripheral blood CD34^+^ cells. After 56 days, 97% of the cells in culture were non-adherent, CD13^+^CD117^+^, toluidine-blue^+^ granular MC (Figure 1).

**Figure 2:**
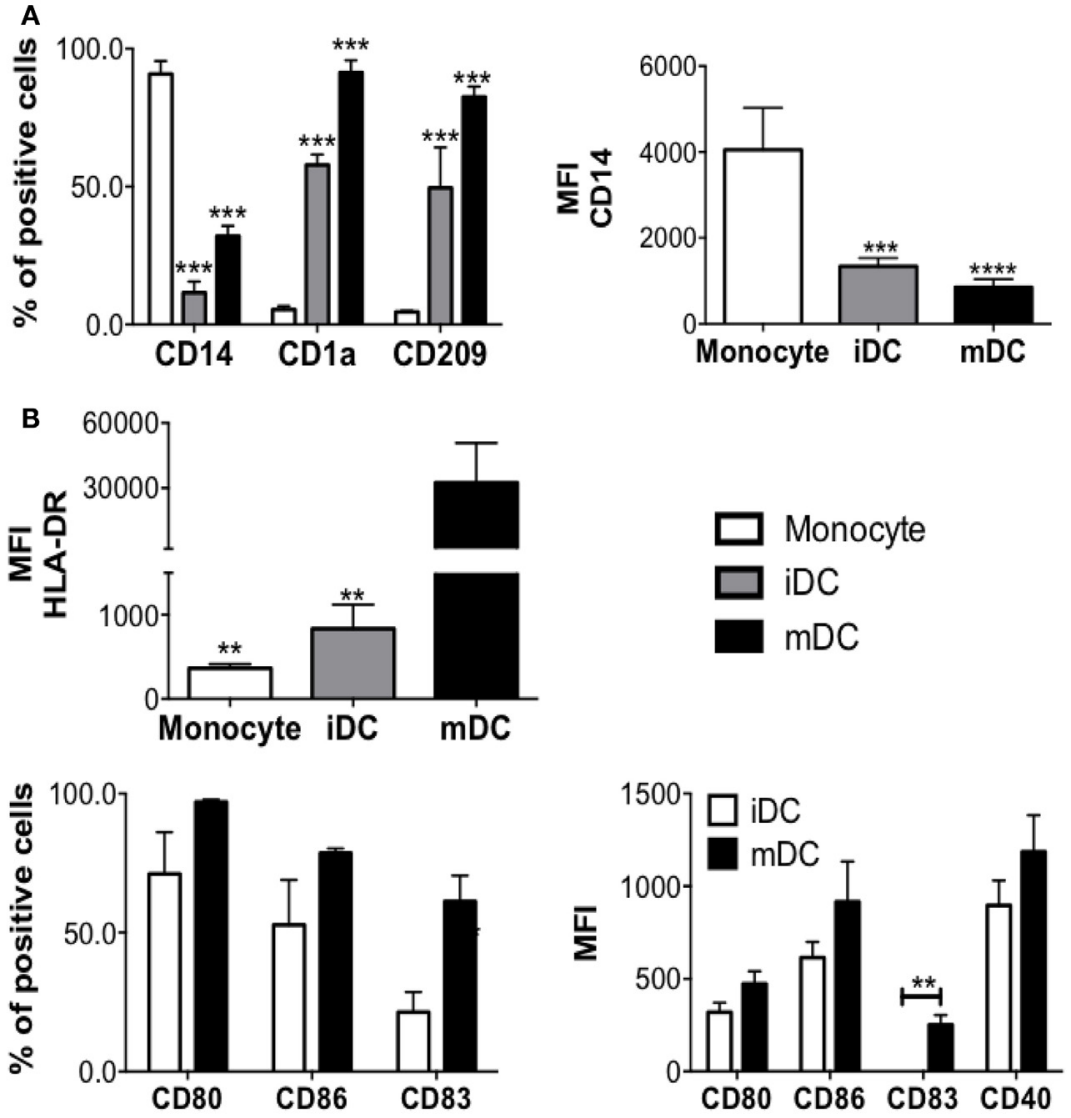
**DC differentiation and maturation**. **(A)** Phenotype changes during DC differentiation (*n* = 4), ***p < 0.001, **p < 0.01 vs. monocytes; **(B)** DCs phenotype changes after DCs maturation induced by TNF-alpha treatment for 48 h (*n* = 4), *p < 0.05 vs. mDCs.

**Figure 3:**
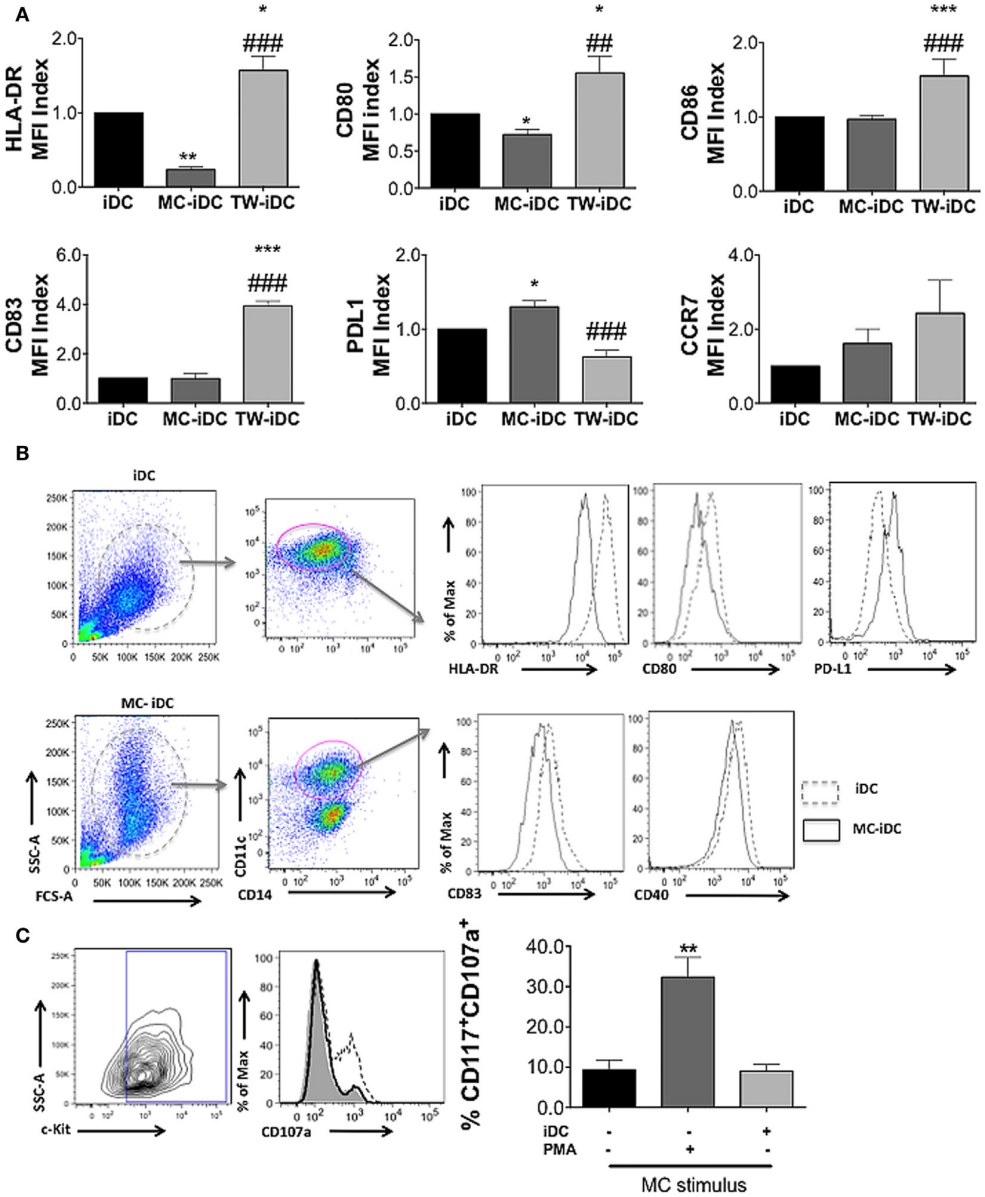
**DC phenotype after coculture with MC**. **(A)** Phenotype of DCs (CD14^−^CD11c^+^ cells) phenotype cultured alone (iDCs), cocultured with MC (MC-iDC), or exposed to MC supernatant in a transwell culture system (TW-iDC); cells were labeled with fluorescent antibodies, the median fluorescence intensity (MFI) was determined for each marker and the MFI index calculated by the formula: MFI of the experimental group/MFI of control iDCs (of each independent experiment, *n* = 4); *p < 0.05, **/^##^*p* < 0.01, ***/^###^*p* < 0.001, * compared to iDC and ^#^ compared to MC-iDC. **(B)** Representative experiment showing the expression of different markers (dotted lines represent iDC and continuous lines, MC-iDC). **(C)** Representative and cumulative data on CD107a expression by MC (CD117^+^ cells) cultured alone (continuous line), in the presence of iDCs (shaded area) or stimulated by PMA (100 nM – dotted line), *n* = 3; **p < 0.01 vs. untreated MC and vs. MC-iDC. All comparisons between groups were performed by one-way ANOVA followed by Tukey.

After MC differentiation was confirmed, PBMC from the same donor(s) were thawed and adherent cells were cultured with IL-4 and GM-CSF for 5 days, to induce their differentiation into DC (Figure 2). iDCs were, then, left alone, cocultured with autologous MC either in direct cell contact (MC-iDC), or separated by a permeable membrane (TW-iDCs) that would allow the passage of soluble mediators or still, stimulated with TNF-α, as a maturation control (mDC) (Figure 2B). After 2 days, the cells were harvested and analyzed by flow cytometry. Compared to iDCs, TW-iDCs displayed increased surface levels of HLA-DR, CD80, CD83, and CD86 (Figure 3A).

Contrastingly, MC-iDC expressed decreased levels of HLA-DR and CD80, had no significant change in CD86, displayed a tendency to increase in CCR7 expression, and significantly higher levels of PD-L1 (Figures 3A,B). As expected, TNF-α induced DC maturation-associated changes in the cell surface phenotype (Figure 2B).

To evaluate if cell contact in MC-iDC culture resulted in MC degranulation, releasing mediators responsible for the DC phenotypic changes, we determined the CD107a expression on MC. MC alone and those cocultured with iDCs displayed the same low levels of CD107a, which, nonetheless, was increased under PMA stimulation (Figure 3C).

### MC-iDC Stimulate Regulatory T Cells

**Figure 4:**
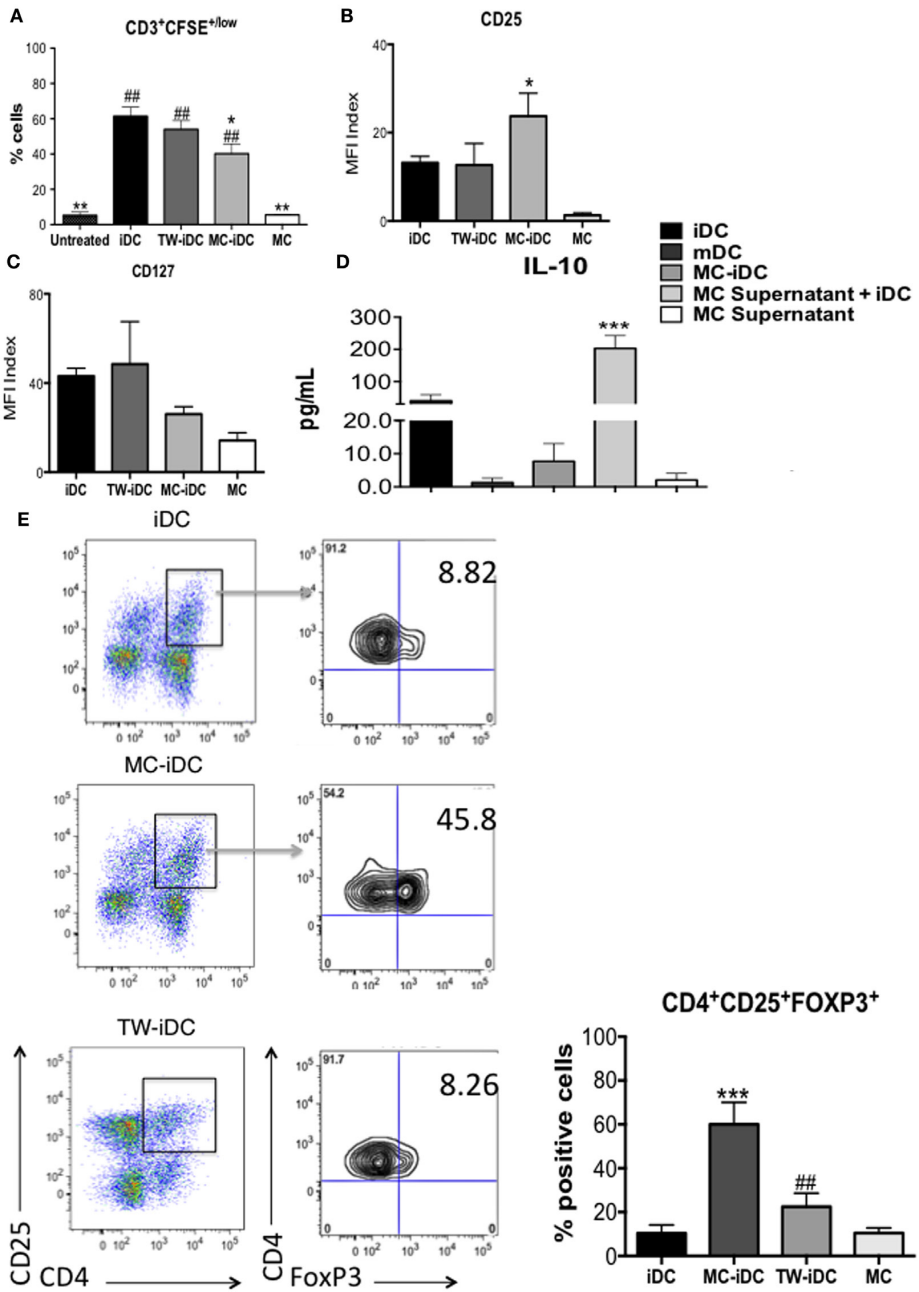
**Induction of Tregs by MC-iDC**. **(A)** Immunomagnetic beads-selected CD3^+^ T cells were labeled with CFSE, stimulated by different cells (iDC *n* = 7; MC-iDC *n* = 8; TW-iDC *n* = 4; MC *n* = 3), at a 10:1 ratio and their proliferation evaluated by dilution of the dye; comparisons were performed by one-way ANOVA followed by the Tukey’s multiple comparisons test, (*) comparisons to iDC and (^#^) comparisons to untreated lymphocytes, */^#^*p* < 0.05, **/^##^*p* < 0.01. **(B)** CD25 expression of T cells stimulated by the various cell populations (iDCs, MC-iDC, TW-iDC, or MC alone); the CD25 expression index was calculated by the formula: (% of positive cells × MFI of positive cells)/1000; *n* = 6. **(C)** The same as **(B)**, but for CD127 expression; *n* = 3. **(D)** Cumulative data on the presence of IL-10 in the supernatant of iDC, DCs treated with TNF-α (mDC), cultured in direct contact (MC-iDC) or with 20% MC supernatant (MC supernatant + iDC) (*n* = 3); MC supernatant alone was used as a control; results assessed by the CBA assay; comparison performed by ANOVA followed by Tukey, ***p < 0.01. **(E)** Representative and cumulative data on FoxP3 expression by CD3^+^CD4^+^CD25^+^ T cells after stimulation with iDCs (*n* = 6), MC-iDC (*n* = 5), TW-iDCs (*n* = 5), or MC (*n* = 7); ***p < 0.001, compared to iDC, ^##^*p* < 0.01, compared to MC-iDC, in one-way ANOVA followed by Tukey.

However, the surface phenotype of DCs is not always predictive of their function (19); so, we evaluated the ability of iDCs, MC-iDC, and TW-iDCs to stimulate allogeneic CD3^+^ T cells. On the one hand, TW-iDCs were not more efficient than control iDCs as T cell stimulators (Figure 4A), despite their increased levels of HLA-DR and CD86 expression. On the other hand, when compared to iDCs, MC-iDC induced lower levels of T-cell proliferation (Figure 4A), which in turn had a significantly higher expression of CD25 (Figure 4B) and a tendency to lower CD127 expression (Figure 4C).

It was intriguing that the increased levels of costimulatory molecules on the surface of MC supernatant-exposed DCs were not paralleled by an enhancement of DC stimulatory activity for allogeneic T cells. The high levels of IL-10 secreted by these DCs might explain this phenomenon (Figure 4D).

**Figure 5:**
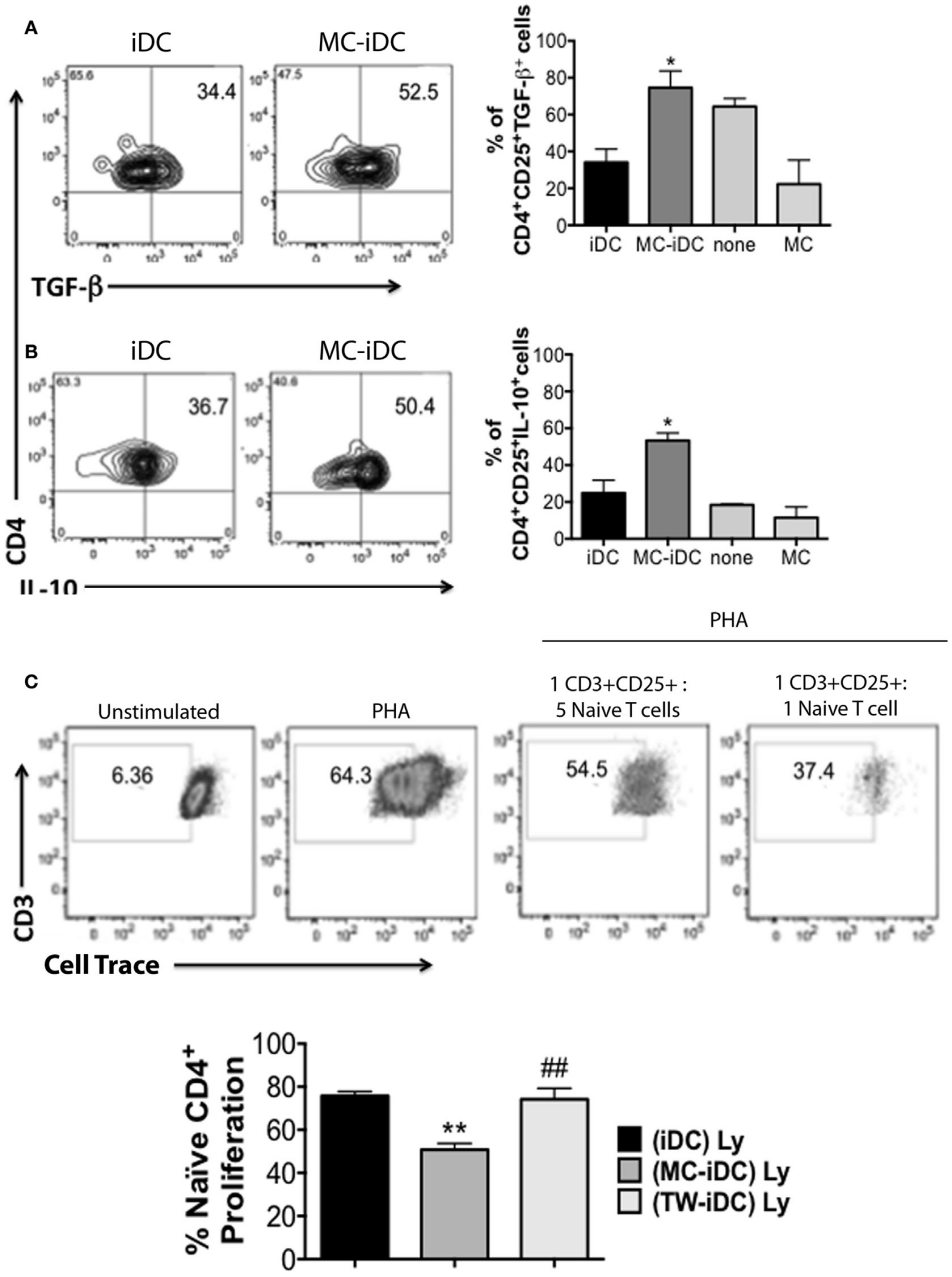
**Cytokine production and suppression of mitogen-induced proliferation of naïve T cells by MC-iDC-stimulated T cells**. **(A)** Representative and cumulative data (*n* = 3) on intracellular TGF-β production by CD4^+^CD25^+^ T lymphocytes submitted to different stimuli (none, or coculture with MC, MC-iDC, or iDCs). **(B)** Representative and cumulative data (*n* = 3) on intracellular IL-10 production by CD4^+^CD25^+^ T lymphocytes submitted to different stimuli (none, or coculture with MC, MC-iDC, or iDCs). **(C)** Representative and cumulative data (*n* = 3) on the effect of MC-iDC-stimulated CD4^+^CD25^+^ T lymphocytes upon mitogen-induced naïve T cell proliferation; stimulated cells were separated and added to violet cell tracer-labeled naïve CD4^+^ T lymphocytes stimulated with PHA (1% v/v, Life Technologies) at different cell ratios and the dilution of the cell tracer determined by flow cytometry, iDC- or TW-iDC-stimulated CD4^+^CD25^+^ were used as controls (cumulative data refer to the 1:1 cell ratio); only living CD3^+^ cells were considered and analyzed by one-way ANOVA, followed by Tukey, *p < 0.05, **p < 0.01 vs. iDC; ^##^*p* < 0.01 vs. MC-iDC.

Furthermore, MC-iDC-stimulated T cells expressed high levels of FoxP3 (Figure 4E) indicating that MC-exposed iDC might be able to induce regulatory T cells (Treg). To confirm this regulatory bias, we analyzed the function of the T cells stimulated by MC-iDC. We noticed that CD3^+^CD4^+^CD25^+^ cells exposed to MC-iDC produced higher levels of TGF-β (Figure 5A) and IL-10 (Figure 5B) and when isolated from the cocultures, suppressed, in a cell dose-dependent manner (Figure 5C), the proliferative response to PHA of naïve T lymphocytes.

### Contact with PD-1 Expressed by MC Induces Expression of IDO by iDCs

**Figure 6:**
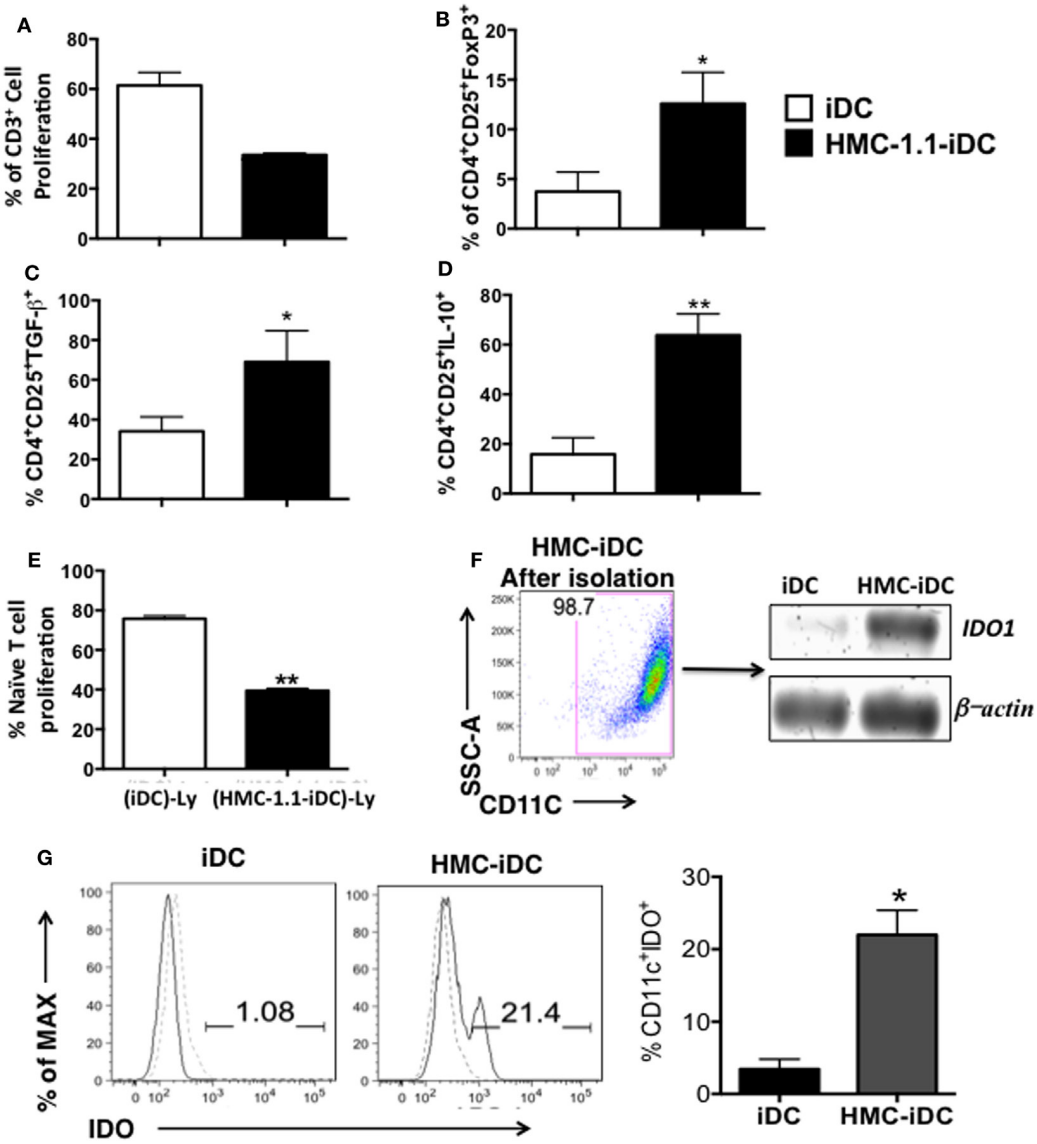
**Also mast cells of the HMC-1.1 induce tolerogenic iDC that show increased IDO expression**. **(A)** Cumulative data (*n* = 3) on the capacity of iDCs cocultured with HMC-1.1 cells (HMC-iDC) to trigger allogeneic T lymphocytes proliferation. **(B)** Cumulative data (*n* = 3) on the expression of FoxP3 in T cells stimulated by iDC or by HMC-iDC. **(C)** Evaluation of the TFG-β or IL-10 **(D)** production by T cells exposed to iDC or HMC-iDC (*n* = 3). **(E)** Cumulative data (*n* = 3) on the effect of HMC-iDC-stimulated CD4^+^CD25^+^ T lymphocytes [(HMC-1.1-iDC)-Ly] compared to control iDC-stimulated cells [(iDC)-Ly] upon mitogen-induced naïve T cell proliferation; DC-stimulated T cells were separated and added to violet cell tracer-labeled naïve CD4^+^ T lymphocytes stimulated with PHA (1% v/v, Life Technologies) in a 1:1 ratio, and the dilution of the cell tracer determined by flow cytometry. **(F)** Representative dot plot from the CD11c^+^ isolation and their expression of *IDO1* by PCR and iDCs were used as control. **(G)** Representative and cumulative data (*n* = 5) on IDO expression by iDCs and HMC-iDC; data were analyzed by Student’s *t*-test, *p < 0.05; **p < 0.01.

IDO expression has been frequently identified as the mechanism through which DCs induce Treg (20). Since, the amount of *in vitro*-differentiated MC would not be enough to execute the next experiments, we evaluated the human MC lineage, HMC-1.1, as a model for MC in our settings. Actually, DCs cocultured with HMC-1.1 (HMC-iDC) displayed the same features as those exposed to CD34^+^ stem cell-derived MC: they were poor triggers of allogeneic T cell proliferation (Figure 6A), stimulated T cells to express high levels of FoxP3 (Figure 6B) and to produce high levels of TGF-β and IL-10 (Figures 6C,D). Finally, such as those cocultured with primary MC, T cells cocultured with HMC-1.1 induced CD3^+^CD4^+^CD25^+^ cells, that when isolated from the coculture, were also able to suppress the naïve T cell proliferation in response to PHA (Figure 6E).

Having determined that HMC-1.1 cells were equivalent to *in vitro*-differentiated MC, we performed the next experiments using this cell line as a model for MC. When evaluating the expression of IDO by iDCs (CD11c^+^ cells isolated from the cultures), we found that when cultured alone, these cells present no mRNA for IDO (Figure 6F) and only very low levels of the protein (Figure 6G). However, when isolated from the coculture with HMC-1.1, they expressed high levels of the message (Figure 6F) and of the protein (Figure 6G).

**Figure 7:**
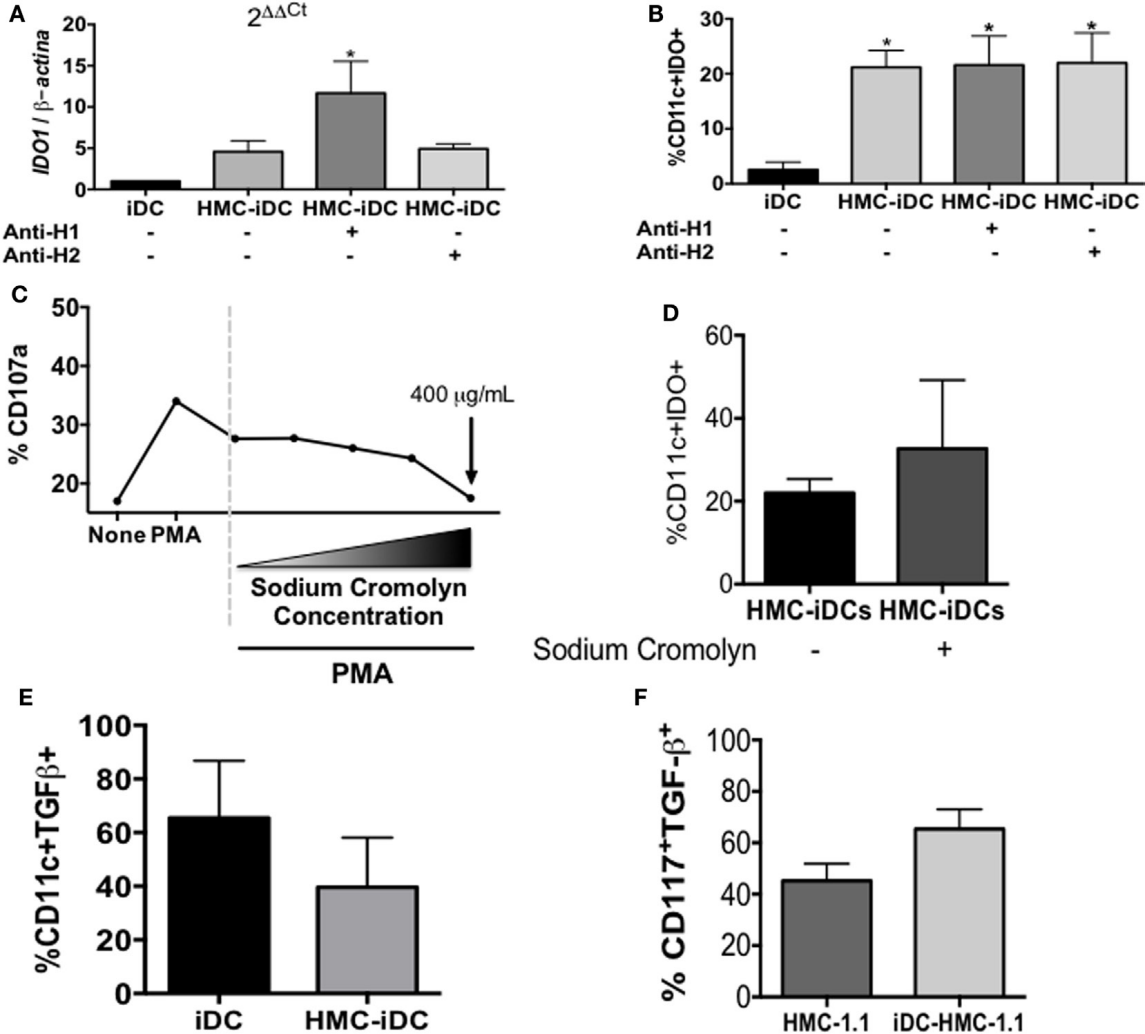
**Blocking of H1 or H2 receptors on iDC did not inhibit IDO expression by HMC-iDC, neither blocked their degranulation**. **(A)** The total RNA of CD11c^+^ cells left alone (iDC), cultured with HMC-1.1 or cultured with H-1 (olopatadine hydrochloride – 50 μg/mL) or H-2 (cimetidine – 50 μg/mL) receptors-blocked HMC-1.1, as indicated by the “+” signal below (*n* = 3). **(B)** Frequency of CD11c^+^IDO^+^ cells, in which iDC were left alone (iDC) (*n* = 5), cultured with HMC-1.1 (HMC-iDC) (*n* = 5), or cultured with H-1 (olopatadine hydrochloride – 50 μg/mL) or H-2 (cimetidine – 50 μg/mL) receptors-blocked HMC-1.1 (*n* = 3), as indicated by the “+” signal below. **(C)** HMC-1.1 were left alone, stimulated with PMA (100 nM) or simultaneously stimulated with PMA and treated with sodium cromolyn at different concentrations (from 3.75 to 400 μg/mL) and their degranulation was evaluated by the expression of CD107a (*n* = 2). **(D)** Sodium cromolyn (400 μg/mL)-treated HMC-1.1 cocultured with iDC still induce increase in IDO expression by the iDCs, which was evaluated after 16 h of coculture by flow cytometry (*n* = 3). **(E)** Frequency of TGF-β^+^ cells among CD11c^+^ cells exposed (HMC-iDC) or not to mast cells (iDC) (*n* = 4). **(F)** Frequency of TGF-β^+^ cells among HMC-1.1 CD117^+^ cells, exposed (iDC-HMC-1.1) or not to iDC (HMC-1.1). Data were analyzed by one-way ANOVA, followed by Tukey, *p < 0.05 vs. iDC.

To evaluate whether the production of IDO was dependent on histamine, we treated the iDC with either an H1-receptor or an H2-receptor blocker before the coculture. Neither blocker affected the expression of IDO by the iDCs, which, again, only expressed the enzyme after coculture with HMC-1.1 (Figures 7A,B).

Furthermore, to exclude the role of other soluble mediators, potentially released by the HMC-1.1 cells (but not likely, since no evidence of degranulation was detected – Figure 3), they were treated with the Ca^2+^ channel-blocker, sodium cromolyn, before coculture. The dose of 400 μg/mL of sodium cromolyn did block HMC-1.1 degranulation (Figure 7C) but had no effect on the ability of these cells to induce IDO expression after coculture with the MC lineage (Figure 7D).

One could argue that the increased production of IDO could be due to an increase in TGF-β in the microenvironment. However, there was no significant increase in either TGF-β^+^ DC (Figure 7E) or TGF-β^+^ HMC-1.1 (Figure 7F) cells after their coculture.

**Figure 8:**
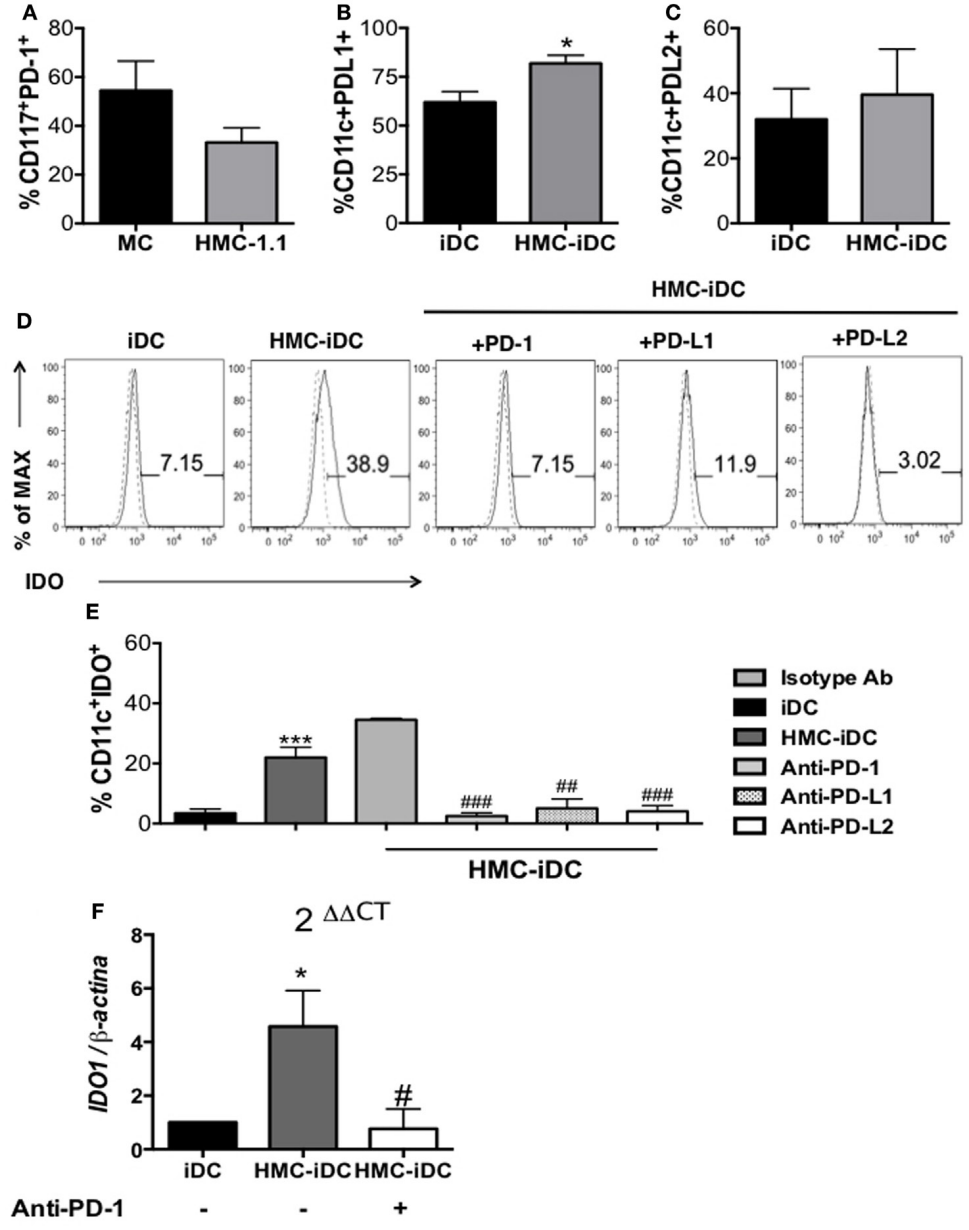
**Mast cells differentiated *in vitro* and the mast cell lineage HMC-1.1 expressed PD-1 and induced increased expression of PD-L1 on iDC**. **(A)** Cumulative data on PD-1 expression by *in vitro*-differentiated MC and HMC-1.1 cells (*n* = 5). **(B)** PD-L1 and **(C)** PD-L2 expression by iDCs and HMC-iDC (*n* = 5); comparison were performed by the unpaired Student’s *t*-test, *p < 0.05 vs. iDCs. **(D)** Representative histogram of the effects on IDO expression by iDCs after coculture with HMC-1.1 under different conditions: blocking PD-1 on HMC-1.1, blocking of PD-L1 or of PD-L2 on iDCs. **(E)** Cumulative data on IDO expression by iDCs (CD11c^+^ cells) cocultured with HMC-1.1 cells; for these cultures, HMC-1.1 were treated or not with anti-PD-1 and iDCs with anti-PD-L1 or anti-PD-L2 (10 μg/10^6^ cells) (*n* = 5); an isotype-matched unrelated mAb was used as control for the treatments. **(F)** qPCR of *IDO1* obtained from the RNA of CD11c^+^ cells isolated by microbeads, from cocultures of iDCs with HMC-1.1 (HMC-iDC) or with anti-PD-1-treated HMC-1.1; data were normalized by β*-actin* expression (iDC *n* = 6; HMC-iDC *n* = 6; HMC-iDC treated with anti-PD-1, *n* = 4); data were compared by one-way ANOVA followed by the Tukey’s *post hoc* test, */^#^*p* < 0.05 and ***p < 0.001 vs. iDCs; ^##^*p* < 0.01 and ^###^*p* < 0.001 vs. MC-iDC.

Among the surface molecules associated with regulation in the immune system, PD-1 was a possible candidate. Its expression by cells of the MC lineage has been reported (21) and was detected on both, our *in vitro*-differentiated MC and on cells of HMC-1.1 lineage (Figure 8A). Furthermore, both PD-1 ligands, PD-L1 and PD-L2, were expressed by the iDCs (Figures 8B,C), which, after contact with MC (both primary and of the lineage), displayed increased levels of PD-L1 (Figures 3A,B and 8B).

Thus, HMC-1.1 cells were treated, or not, with anti-PD-1 before coculture with iDC and the phenotype of the resulting HMC-iDC determined. Anti-PD-1 treatment of HMC-1.1 inhibited their ability to induce IDO^+^ iDCs (both at protein and *IDO1* mRNA levels) (Figures 8D,F). And confirming the role of this molecule, the treatment of iDCs with either anti-PD-L1 or anti-PD-L2 also inhibited IDO expression (Figure 8E).

### PD-1 Induces IDO Expression on iDCs Probably *via* STAT-3 Signaling

**Figure 9:**
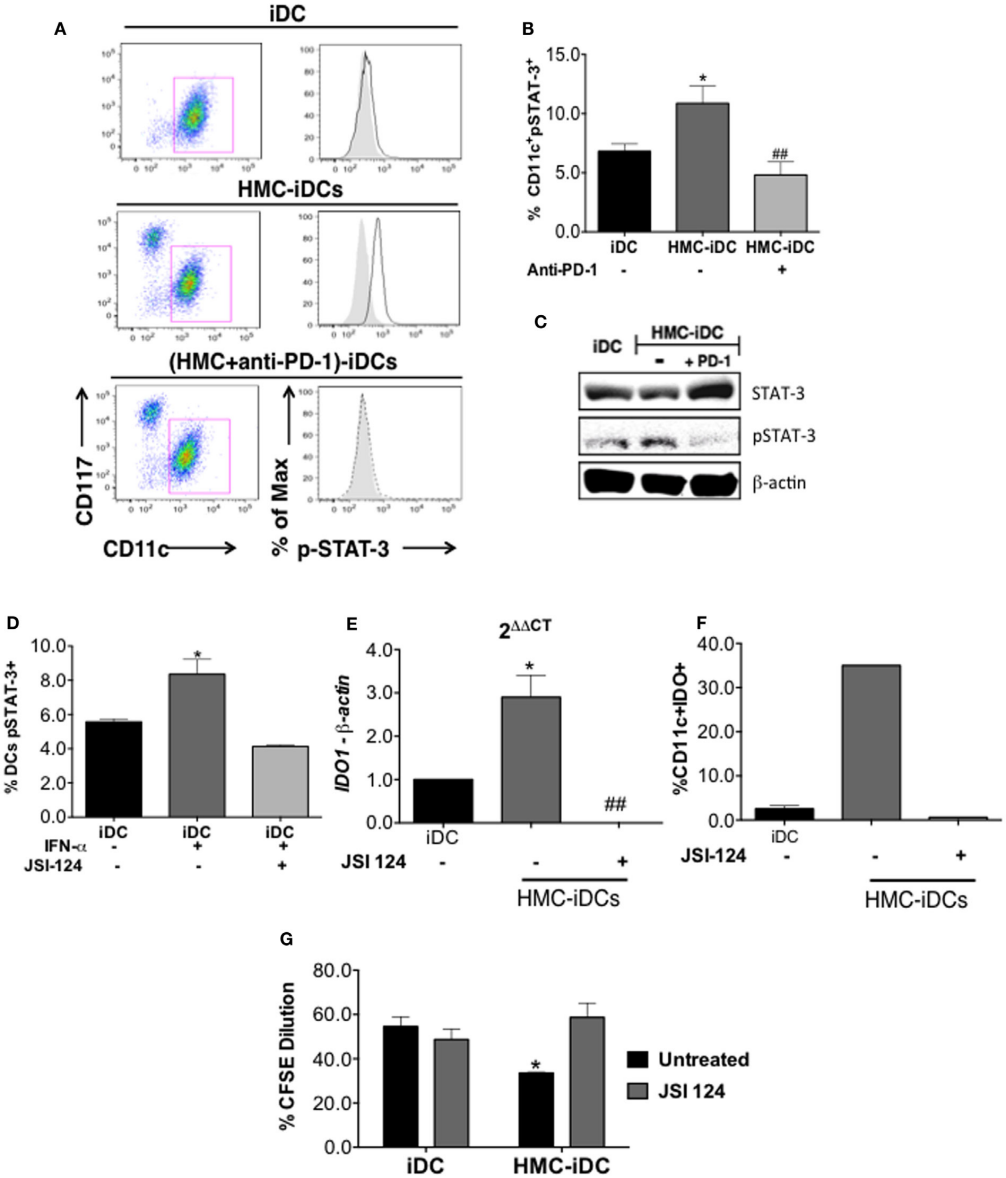
**Coculture with HMC-1.1 induced increase in p-STAT-3 content of iDCs and was prevented by anti-PD-1 treatment of HMC-1.1**. **(A)** Representative dot-plot showing the increase in p-STAT-3 content of CD11c^+^ cells (iDCs) after coculture with HMC-1.1. **(B)** Cumulative data on the frequency of CD11c^+^p-STAT-3^+^ cells among iDCs (*n* = 5), HMC-iDC (*n* = 4), and (anti-PD-1-treated HMC-1.1)-iDCs (*n* = 4); comparison was performed by one-way ANOVA followed by Tukey, *p < 0.05 compared to iDCs, ^##^*p* < 0.01 compared to HMC-iDC. **(C)** Immunoblot for p-STAT-3, STAT-3, and β-actin from iDC, HMC-iDC, or iDC cultured with HMC-1.1 treated with PD-1 (HMC-iDC + PD-1). **(D)** JSI-124 (0.5 μM) inhibited p-STAT-3 expression by IFN-α (20 ng/mL)-stimulated iDC. **(E)** qPCR of *IDO1* from the RNA of CD11c^+^ cells, CD11c^+^ cultured with HMC-1.1 (HMC-iDC), or with iDC treated with JSI-124 (0.5 μM) and cultured with HMC-1.1 (*n* = 3), the CD11c^+^ were isolated by microbeads. **(F)** Frequency of IDO^+^CD11c^+^ cells detected by flow cytometry among iDCs or among iDC cultured with HMC-1.1 (HMC-iDC), treated or not with JSI-124 (0.5 μM) (*n* = 3). **(G)** CD3^+^ T cell proliferation induced by the different cell populations (iDC *n* = 6; iDC treated with JSI-124, *n* = 3; HMC-iDC, *n* = 3; HMC-iDC exposed to JSI-124, *n* = 3); comparison among the groups was performed by one-way ANOVA followed by Tukey; (*p < 0.05) compared to iDC and (^##^*p* < 0.01) compared to HMC-iDC.

**Figure 10:**
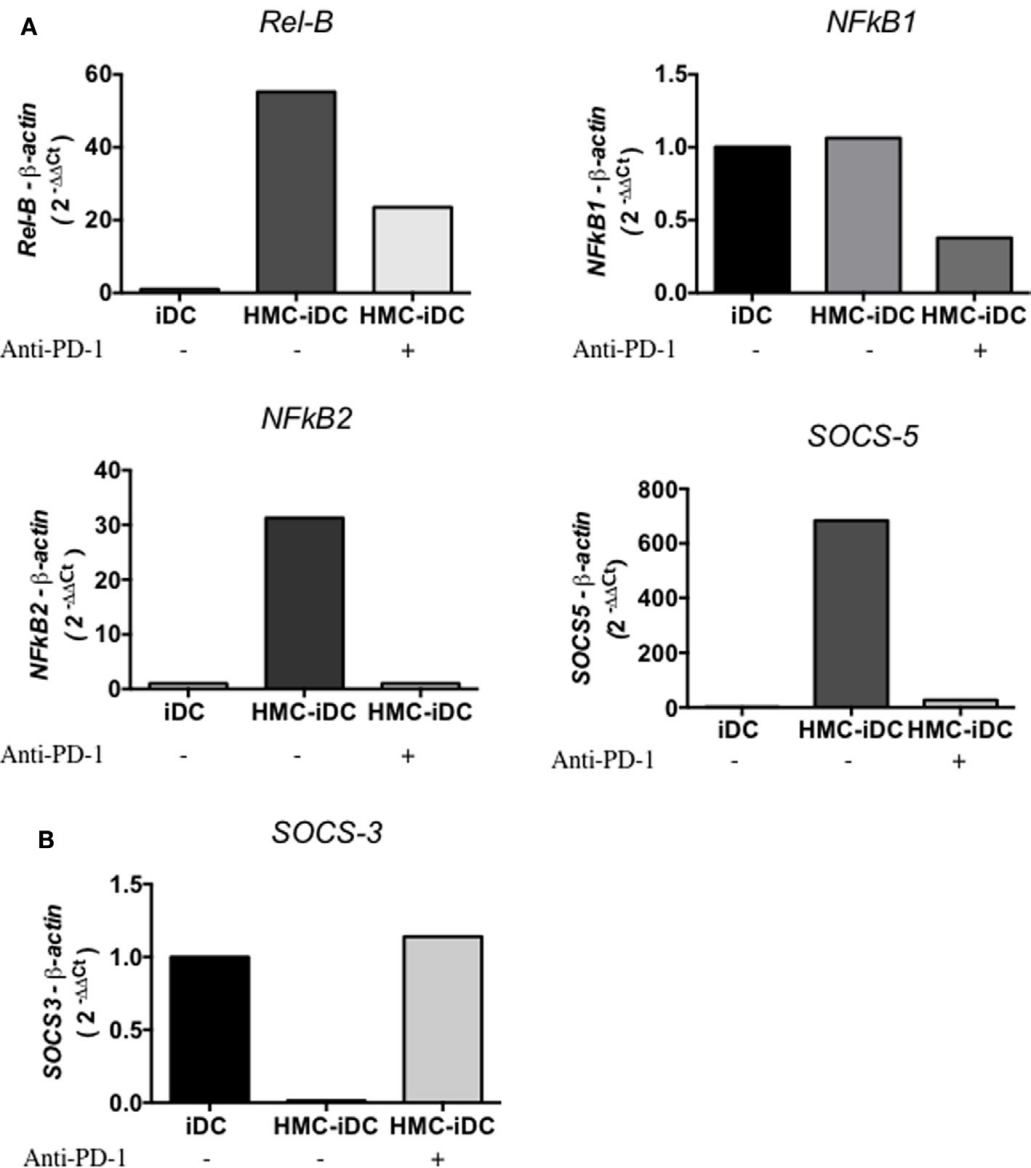
**Expression of p-STAT-3 modulated genes by iDCs and HMC-iDC**. HMC-1.1-exposed iDC isolated by CD11c^+^ microbeads demonstrated an increased expression of *Rel-B*, *NFkB1*, *NFkB2*, and *SOCS5* and a decreased expression of *SOCS-3* that could be blocked by anti-PD-1 treatment of HMC-1.1 cells. **(A)** After 16 h of coculture, CD11c^+^ cells exposed to HMC-1.1 or HMC-1.1 treated with anti-PD-1 were isolated, had their total RNA extracted using TRIzol, and the expression of *Rel-B*, *NFkB1*, *NFkB2*, and *SOCS5* were determined. **(B)** After 2 h of coculture, we evaluated the expression of *SOCS-3* from the isolated CD11c^+^ cells.

IDO production by DCs is positively regulated by STAT-3 phosphorylation (22) that is, also, a positive regulator of the non-canonical NF-kB pathway (22, 23). Coherently, the coculture of iDCs with HMC-1.1 cells, increased STAT-3 phosphorylation on iDCs, but the same did not occur when HMC-1.1 were treated with anti-PD-1 (Figure 9) This was detected by flow cytometry, which showed an increased frequency of cells with phosphorylated STAT-3 (pSTAT-3) among HMC-iDC (Figures 9A–C) and confirmed by western blot (Figure 9D). Furthermore, it has been reported that p-STAT-3 enhances the expression of Rel-B, NFkB1, NFkB2, and SOCS5 (22, 24) genes whose enhanced expression was also detected in HMC-iDC (Figure 10A). On the one hand, supporting the role of PD-1 as the trigger of an enhanced phosphorylation of STAT-3 in the iDCs, we noticed that blocking of PD-1 inhibited the enhanced expression of the genes (Figure 10A). On the other hand, the level of SOCS-3 in iDCs was decreased after 2 h of coculture with HMC-1.1 (Figure 10B), a phenomenon that was blocked by treatment of the HMC-1.1 line with anti-PD-1. This last observation is coherent with the reported ability of SOCS-3 to inhibit p-STAT-3 (25) and IDO (26).

Finally, to assess, through a different strategy, the role of pSTAT-3 in the induction of IDO^+^ DCs, we treated the iDCs, before exposing them to the HMC-1.1, with 0.5 μM cucurbitacin (JSI-124), an inhibitor of the STAT-3 pathway (22), as shown in Figure 9D. After 16 h of JSI-124 exposure, the DCs were analyzed for their IDO expression (by qPCR and flow cytometry), and a significant decrease in their IDO content was found (Figures 9E,F). Furthermore, these JSI-124-treated HMC-iDC recovered the ability of untreated iDCs to stimulate allogeneic T cell proliferation (Figure 9G).

## Discussion

The data presented here show that MCs, much more than being the culprits of acute allergic reactions (27), are able to modulate the function of DCs *via* direct cell contact. Though roles for MC beyond those which they play in allergic reactions have been recently suggested (28), most reports ascribe these regulatory functions to MC soluble products (28–32). The data presented here, however, indicate that, in our experiments, MC bend DCs toward a tolerogenic function *via* cell membrane contact between PD-1, on MC, and its ligands, PD-L1 and PD-L2, on DCs.

Mast cell soluble products have been shown to affect DCs toward a proinflammmatory function (9, 32, 33), and also we noticed that DCs, exposed to MC soluble products, through a transwell culture system, show phenotypic changes that suggest their maturation. These DCs expressed higher levels of CD86, CD83, and HLA-DR.

Interestingly, though, in our experiments, the increased levels of costimulatory molecules on the surface of MC supernatant-exposed DCs were not paralleled by an enhancement of DC stimulatory activity for allogeneic T cells. This could be explained by the high levels of IL-10 secreted by these DCs and is coherent with data from the literature showing that MC significantly reduced inflammatory reactions in graft-versus-host disease and in infections, by an IL-10-dependent mechanism (34, 35). Thus, tissues rich in MC, such as the mucosa, would be partially protected from immune-inflammatory damage, by DC-derived IL-10, secreted as result of their exposure to MC soluble mediators in a regulatory feedback among MC, DCs, and T cells.

Contrastingly, the surface phenotype of DCs cultured in direct contact with MC was characterized by a decreased expression of costimulatory molecules and, notably, an increased expression of the coinhibitory molecule, PD-L1. This molecule has a relevant role in controlling T cell responses through its interactions with PD-1 on T lymphocytes (36), but little is known about the consequences of its interactions on the DCs themselves, which express both PD-L1 and PD-L2 (37).

Though the phenotype of MC-iDC suggested a poor T-cell stimulating ability, the surface phenotype of DCs is not always predictive of their function (19). So, in order to assess if the phenotype of MC-iDC corresponded to a decreased stimulatory function, we tested their ability to induce the proliferation of allogeneic T cells, and it was, in fact, poor. Not only that, MC-iDC-stimulated T lymphocytes were actually regulatory T cells that showed an increased expression of CD25 and FoxP3, produced high levels of IL-10 and TGF-β, and, when isolated from the cocultures and added to naïve T cells, inhibited their response to PHA. Though we did discriminate between the expansion of already present Tregs and the differentiation of new cells, their actual suppressive ability suggests that they might have significant impact on immune responses occurring in their presence. Actually, this resembles what was already described in mice (9), where MC bias DCs to become stimulators of Tregs.

At this point, another issue comes to one’s attention. We show that MCs affect the phenotype and function of immature DCs, which, usually, remain in the tissues and, thus, should have less impact upon the immune response than mature DCs (38). However, one should consider that still immature DCs entering a tissue rich in MC might be biased into such “tolerogenic” status, which could, in turn, hamper local immune responses. Furthermore, it is worth mentioning that even though MC-iDCs had decreased levels of various maturation-associated molecules, these cells had a marginally enhanced CCR7 expression. This expression might increase their migratory potential, allowing them to reach lymphoid organs and affect the development of T cell responses *in vivo*. However, Dudeck et al. (39) have recently shown a clear positive impact of MC upon DC maturation and migration. In a mouse system, the authors demonstrate that MC-derived TNF is critical for the maturation of CD8^+^ DCs and for the activation of contact allergens-specific CD8^+^ T cells. Indeed, though apparently contradictory, these data are in agreement with the already observed ambiguous role for MC in the modulation of immune responses, where they have been shown to either stimulate or suppress, both innate and acquired immune responses (40).

We also noted that MC did not degranulate after contact with DCs. This is coherent with the described effect of mouse MC upon Tregs induction, which can be inhibited by MC degranulation (41).

It is worth noting that MCs have been described as modulating the T cell response patterns, sometimes favoring a Th2 profile (42, 43), but also of Th1 (44) and Th17 (45). However, this modulatory activity has been consistently associated with MC degranulation, which we did not detect, whereas the effects we describe were induced by direct cell contact between MCs and DCs.

Actually, the induction of Tregs by MC has been already reported (9) and is in agreement with the tolerogenic activity of MC in transplantation models (10). What our observations add, however, is the indication that the stimulation of Tregs by MC may not be the result of direct MC-T cell interaction, but a consequence of the bias induced on DC by the MC.

Intriguingly, although the induction of Tregs by DCs has been reported to be dependent on IL-10 (46, 47), this does not seem to be the case here. Though DCs exposed to MC supernatants did secrete IL-10, they did not bias T cell differentiation toward the regulatory phenotype – while those that had direct contact with the MC did induce Tregs, but did not secrete IL-10. In our experiments, in fact, the induction of Treg seemed to be dependent on IDO expression by the DCs, an enzyme whose expression has been associated with Treg induction (48) and tolerance in a wide range of settings, from maternal–fetal interactions to cancer (20).

Though HMC-1.1 cells, the MC lineage we used, are poor producers of granules and histamine (49) and our data suggested that soluble products were not responsible for the modifications on DCs cocultured with MCs, we investigated if histamine, which has been shown to induce IDO expression by DCs (50), was involved in their response to MC. We treated DCs with blockers of H1 (olopatadine hydrochloride) and H2 (cimetidine) receptors and HMC-1.1 cells with the Ca^2+^ inhibitor, sodium cromolyn, and evaluated IDO expression by the DCs. As expected, none of these treatments decreased IDO expression by HMC-iDC.

Hence, the modification of DCs by contact with MC had to be explained by other mechanisms. Among these, TGF-β has been associated with the long-term induction of IDO in DCs (23, 51), which under the influence of this growth factor extend their regulatory functions to other cells (51, 52). However, blocking the TGF-β receptor on DCs with anti-CD105 monoclonal antibodies did not affect their IDO expression after coculture with MC. Though we cannot exclude a role for TGF-β (which was present in coculture supernatants – Figure S1 in Supplementary Material) in the suppressive milieu, this observation suggests that the triggering of IDO production in HMC-iDC is not dependent of this cytokine.

Another eliciting factor for IDO expression by DCs is recombinant soluble CTLA-4 (53), a ligand of B-7 costimulatory molecules. CTLA-4, however, is not expressed by MC (6), but, PD-1, another B-7 ligand is (21). And PD-1 interaction with PD-L1 or PD-L2 also has been shown to induce immunosuppression, either by causing T cell anergy or regulatory phenotype (37). We show here that this interaction can have another consequence: when occurring between MC and iDCs, it can induce IDO expression by the latter, since HMC-1.1 treated with blocking antibodies against PD-1 had their ability to induce IDO expression by iDCs abolished. Coherently, the blocking of PD-L1 and PD-L2 on iDCs was able to decrease their IDO expression.

Furthermore, HMC–iDC interaction caused an increase in p-STAT-3 in DCs, which was blocked by anti-PD-1, an observation that agrees with the known control of IDO expression by the non-canonical NF-kB pathway (23), activated by p-STAT-3 (22).

Also, after interaction with HMC-1.1, we noticed an increase in *SOCS-5*, *RelB*, *NFkB1*, and *NFkB2* and a decrease in *SOCS*-*3*, phenomena that are coherent with the activation of p-STAT-3 in the cells (24, 25). Finally, we blocked STAT-3 activation on DCs using an inhibitor, JSI-124, which enhances the canonical but impairs the non-canonical NF-kB pathway (22). JSI-124-treatment of DCs prevented the phenotypic changes induced by coculture with HMC-1.1 and the treated iDCs, maintained their capacity to stimulate T cell proliferation.

In conclusion, these findings show that MC can affect iDCs inducing, by direct cell contact, phenotypic and functional changes that favor the activation of Tregs. This bias is associated with an increase in IDO expression by the iDCs, which depends on the interaction of PD-1 (on MC) with its ligands (PD-L1 and/or PD-L2 on DCs) and which seems to be mediated by an increase in p-STAT-3 in DCs.

## Author Contributions

CR performed the research, collected and interpreted data, and wrote the manuscript. AF helped to discuss some results. MPM helped to perform the qPCR. CM helped to perform the JSI-124 experiment. PB-S helped to discuss some results. JB designed the research, discussed the results, wrote the discussion, and reviewed the manuscript.

## Conflict of Interest Statement

The authors declare that the research was conducted in the absence of any commercial or financial relationships that could be construed as a potential conflict of interest.
