# Supplementary figures and images for: Tolerogenic IDO+ Dendritic Cells Are Induced by PD-1-Expressing Mast Cells
Source: Front Immunol. 2016 Jan 25;7:9. doi: 10.3389/fimmu.2016.00009 (PMC4724729; doi:10.3389/fimmu.2016.00009)

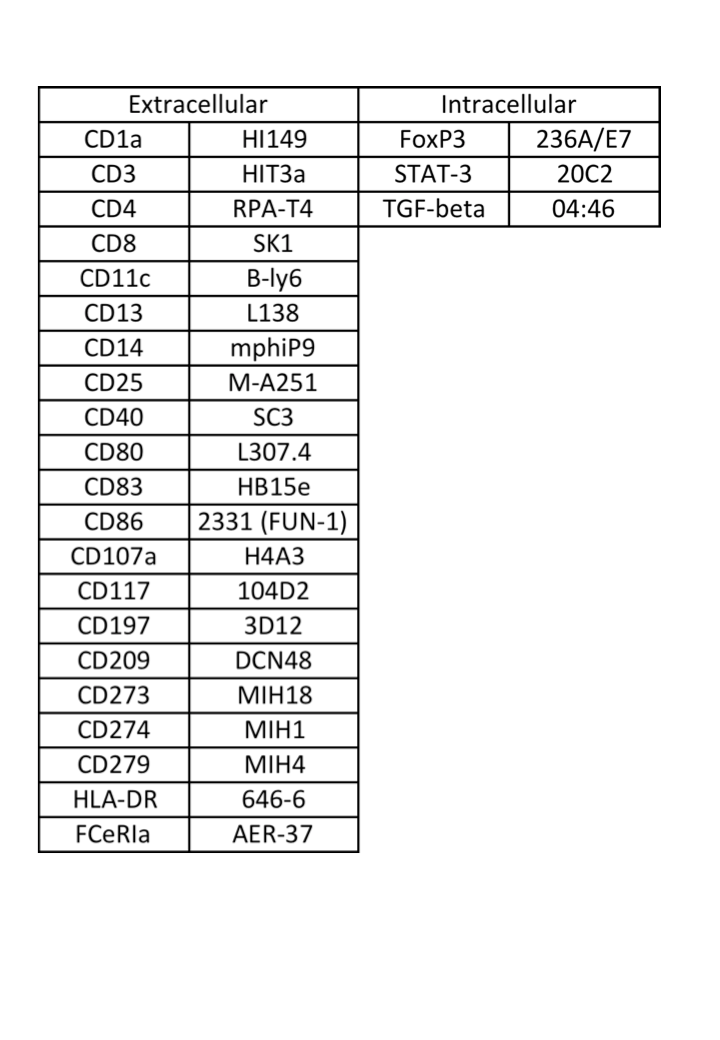

Supplement: Supplementary file 1 [file Image_1.TIFF]
